# The challenges of midlife women: themes from the Seattle midlife Women’s health study

**DOI:** 10.1186/s40695-018-0039-9

**Published:** 2018-06-15

**Authors:** Annette Joan Thomas, Ellen Sullivan Mitchell, Nancy Fugate Woods

**Affiliations:** 1 College of Nursing, Seattle University, Seattle, Washington USA; 2 Family and Child Nursing, University of Washington, Seattle, Washington USA; 3 Biobehavioral Nursing and Health Informatics, University of Washington, Seattle, Wahsington USA

**Keywords:** Midlife women, Challenges, Multiple co-occurring stressors, Divorce, Health concerns, Deaths of parents, Parenting

## Abstract

**Background:**

Midlife, the period of the lifespan between younger and older adulthood, has been described as a period of transition in women’s lives. Investigators studying midlife have focused on women 40 to 65 years of age, who typically experience multiple social, psychological and biological challenges, among them the menopausal transition. Investigators have reported a diverse array of stressful events, for example, health concerns, family problems, work-related issues, deaths, frustrated goal attainment, and financial worries; however, none have identified which life events midlife women experience as the most salient. The purpose of this study was to understand the meaning behind the experiences that midlife women identify as the most challenging.

**Methods:**

Participants were enrolled in The Seattle Midlife Women’s Health Study, a longitudinal study spanning up to 23 years. Summative content analysis, incorporating manifest and latent analysis approaches, was used to identify life experiences that women described as the most challenging looking back over 15 years of being in the study. Eighty-one women responded to the question, “Since you have been in our study (since 1990 or 1991), what has been the most challenging part of life for you?”

**Results:**

Women identified the most challenging aspects of midlife as changing family relationships, re-balancing work/personal life, re-discovering self, securing enough resources, and coping with multiple co-occurring stressors. Within these themes the most frequently reported challenges were: multiple co-occurring stressors, divorce/breaking up with a partner, health problems of self, and death of parents. Few women mentioned menopause as the most challenging aspect of their lives.

**Conclusion:**

Women found themselves searching for balance in the midst of multiple co-occurring stressors while coping with losses and transitions, for some in a context of limited resources. Menopause was infrequently mentioned. Future research to identify the challenges experienced by more diverse populations of women and further understanding of the dynamics among multiple co-occurring stressors is needed to provide individualized health care appropriately to midlife women.

## Background

Midlife, the period of the lifespan between younger and older adulthood, has been described as a period of transition in women’s lives. Investigators studying midlife have focused on women 40 to 65 years of age, who typically experience multiple social, psychological and biological transitions. Among these are the biological transition of menopause, developmental transitions related to the aging/emerging self, and situational transitions such as divorce, taking on caregiving responsibilities for parents, or launching children [1].

In an investigation of the meaning of midlife, Woods and Mitchell [2] found that participants in the Seattle Midlife Women’s Health Study described experiencing a diverse array of stressful events, for example, health problems, family problems, work-related issues, deaths, frustrated goal attainment, and financial concerns [2]. Women reported health problems they were experiencing as well as those their parents experienced with similar frequency. Deaths were also a common experience for these women. Family challenges included challenges with adolescent children, domestic violence, divorce or separation from a spouse, and the ending of relationships. Work problems included difficulty finding work, workplace conflicts, and downsizing of workplaces. These midlife women also reported frustrated goal attainment, such as being unable to complete an academic program or lack of personal time while working. In addition, they experienced financial stresses such as inability to pay college tuition for a child or afford essentials. SMWHS participants also rated their perceived stress levels in a health diary throughout their participation the study [3]. Greater perceived stress levels were significantly related to employment, history of sexual abuse, depressed mood, negative appraisal of aging changes, and poorer perceived health. Although symptoms such as hot flashes, sleep disruption, difficulty concentrating, and depressed mood were associated with greater perceived stress, the menopausal transition, itself, was unrelated to perceived stress. Improvement in role burden, social support, and income adequacy were associated with significantly lower perceived stress levels. Although SMWHS participants reported stressful experiences during midlife and rated their stress levels in a variety of dimensions of their lives over an extended period of time, it was not clear which stressors were most salient to them.

One approach to understanding experiences midlife women find most salient is inquiring about challenges they face. “Challenges” refer to experiences that require great physical or mental effort and determination that test one’s strength, skill, or ability. In comparison, a stressor is a stimulus or threat that places real or perceived demands on the body, emotions, mind, or spirit of an individual. The word challenge, used in this study, is a word embroidered with strength and courage, allowing for the possibility that all challenges may not be perceived as stressful or appraised as negative. Due to the design of the original survey instrument, the words “challenge” and “stressor” as well as “challenging” and “stressful” were interchangeable in this report. In recent studies, women have reported challenges due to racism such as derogatory remarks, discriminatory actions such as sexual harassment [4] as well as menopausal symptoms, such as forgetfulness or difficulty concentrating (cognitive function), mood disturbances, and sleeping problems [3].

A commonly reported challenge of midlife is managing multiple responsibilities attributable to women’s multiple roles. During midlife many women have been married or partnered, have already had children (some are young, others are leaving for college or jobs), have jobs of their own, manage their household with or without any additional help, and care for their aging parents. Kenny [5] studied stressors reported by 299 women aged 18–66 years and found that midlife women had more stressors than younger or older women and that midlife women identified roles involving family, work, and eldercare as sources of stress [5], but did not identify which of these sources of stress was the most salient.

During midlife, some women experience severing a relationship with a long-term partner. In addition to being emotionally wounded, women may experience a substantially reduced household income. Women in midlife tend to experience higher rates of loneliness and distress post-divorce than do younger divorced women [6].

Women in midlife begin to experience health problems of their own, such as cardiac problems [7] and sleeping difficulties [8]. Evidence from recent studies suggests that some of these health problems are related to women’s stressful experiences. Investigators for the Study of Women’s Health Across the Nation (SWAN) sleep study, examined very stressful life events using an 18-item version of the Psychiatric Epidemiology Research Interview Life Events Scale (PERI), which evaluated eight areas: school, work, romantic relationships, children, family, criminal and legal matters, finances and health. Women who had high chronic stress levels had lower subjective sleep quality, more waking after sleep onset (WASO) and were more likely to report insomnia compared to women who had low to moderate chronic stress profiles [8].

Allostatic load has been proposed as the accumulation of stress over time that affects health leading to preclinical signs of disease [9]. Allostasis refers to the ability to achieve stability and to maintain homeostasis during changing conditions through physiological or behavioral means and is adaptive in the short-term [10] but can revert to chronic stress in the long-term (allostatic load). Some events in daily life can generate chronic stress resulting in “wear and tear” on a woman’s body, resulting in allostatic load [11]. Physiologic responses to stress are mediated by epinephrine and/or cortisol, which in turn elevate heart rate and blood pressure. Constant elevation of these responses over time can result in atherosclerosis increasing the risk for myocardial infarction and stroke [10]. Thus, health problems and allostatic load may be a result of chronic exposure to stressors or challenges.

Some of the questions that remain unanswered about midlife women’s experiences of stress include: *Which life events do midlife women experience as the most stressful? Which of these life events are the most challenging to midlife women?*

Although studies of midlife women have documented multiple sources of stress, the impact of stressful life events and perceived stress on symptoms, subclinical changes, and diagnosed disease such as cardiovascular disease, to date there are no studies that reveal the most salient challenges for midlife women. The purpose of this study was to identify the experiences that midlife women found most challenging as they reflected on their experiences over more than a decade of their lives.

## Methods

### Study design and population

Data reported here were collected as part of a longitudinal study, the Seattle Midlife Women’s Health Study (SMWHS). Women entered the study between 1990 and the early part of 1992 when most were in the early stages of the menopausal transition (MT) or not yet in the transition. All households within census tracts with a wide income range and mixed ethnicity were contacted by telephone for interested and eligible women. Women who were eligible were between 35 and 55 years of age, had at least one menstrual period within the last year, had a uterus and at least one ovary, were not pregnant, and could read and understand English. Out of 11,222 telephone contacts, 820 women were eligible, and 508 women entered the study [12].

The University of Washington Institutional Review Board approved each phase of the Seattle Midlife Women’s Health Study (SMWHS) and approved informed consent forms. Each participant signed informed consent forms before entering the study.

Women completed an initial in-person interview administered by a trained registered nurse interviewer. A subset of the 508 women kept a health diary and from late 1996 to 2005 provided urine samples. All women were mailed a yearly Health Questionnaire and kept a menstrual calendar.

### Sample

Eligible participants for this study (*N* = 81) provided data from the 2006 Health Questionnaire answering specifically the following question: “Since you have been in our study, what has been the most challenging part of life for you?” A total of 83 women responded to the 2006 Health Questionnaire. Two women did not answer this specific question leaving 81 women’s answers for analysis. Women not eligible for this study did not answer the 2006 Health Questionnaire.

At enrollment in the parent study, women who were eligible for inclusion were midlife women with a mean age of 39.3 years (SD 3.0 years) and in the current study were approximately 53–54 years, an education of 16.6 years (SD 2.7 years), and mean family income of $38,320 (SD $14,782). Most (86%) were employed. Eligible women described themselves as African American (3.6%), Asian/Pacific Islander (8.3%), or White (88.1%). Women eligible for this study identified themselves as never married or never partnered (6%), married or partnered (76.2%), divorced or separated (16.7%) and widowed (1.2%). Most (67%) of the eligible women were parents.

As seen in Table 1, women with data included in the current analyses compared to those who were ineligible were similar with respect to family income, employment status, and marital status. They differed significantly by age, years of education, and race/ethnicity; women in the current study were younger, had more years of education, fewer were African American and more were White women, and fewer women were parents.

**Table 1:** Sample Characteristics at Start of Study (1990–1991) for the Eligible and Ineligible Women in the Challenges of Midlife Women from the SMWHS

Characteristic	Eligible *N* = 81	Ineligible *N* = 427	*p* value^a^
	Mean (SD)	Mean (SD)	
Age (years)	39.3 (3.0)	42.2 (4.7)	<.0001
Years of Education	16.6 (2.7)	15.5 (2.9)	<.0017
Gross Family Income	38, 320 (14,782)	35, 460 (15,258)	= .1210
Characteristic	% (N)	% (N)	*p* value^b^
Currently Employed	86%	86.3%	= .9428
Race/Ethnicity % (N)			
African American	3.6% (3)	13% (55)	= .0152
Asian/Pacific Islander	8.3% (7)	8.5% (36)	= .9528
White	88.1% (74)	74.8% (317)	= .0093
Other (Latina/Hispanic, Mixed/NativeAmerican)		3.8%	= .0749
Marital status % (N)			
Never married/partnered	6% (5)	7.1(30)	= .7211
Married/partnered	76.2% (64)	67.0% (284)	= .1028
Divorced/separated	16.7% (14)	24.1% (102)	= .1469
Widowed	1.2% (1)	1.9% (8)	= .6634
Ever a parent?			
Yes	60.7%	72.9%	0.0268
No	26.2%	27.1%	0.8673

^a^independent t-test

^b^Chi-square test

### Analysis

Summative Content Analysis approach as described by Hsieh and Shannon [13] was used to identify life experiences that midlife women described as challenges looking back over 15 years when they participated in the SMWHS. Summative content analysis is a method that researchers use to interpret the content of data through coding in order to identify themes about the study participants’ life experiences. Consistent with the approach, content analysis of data in this study started with identifying and quantifying key words or phrases in the text with the purpose of understanding the contextual use of the words or phrases. Some of the key words and categories that were identified prior to analysis were derived from the categories of the Life Event Questionnaire [14] while others were newly identified from the text. Potter and Levine-Donnerstein [15] refer to this first level of analysis as manifest content analysis whereby the appearance of the particular word or content is analyzed rather than the meaning derived. Each response was read over initially for a first impression. Subsequent readings included circling of key words or phrases in the women’s answers in order to develop a coding scheme. The women’s responses were divided into five themes with categories listed under each main theme. The responses were listed under the appropriate category and ranged from one sub-category to five sub-categories, meaning that the women identified from one to five types of challenges. Disagreements about coding the challenges were discussed among the investigators (AJT, NFW) until a resolution was found.

In this study, counting of key words and phrases enabled the authors to identify patterns in the data and to contextualize the codes, which subsequently led the authors to discover meanings, a process which Morse and Field [16] refer to as latent content analysis, an aspect of the summative content analysis approach. Credibility or internal consistency of findings was assured by aligning the textual evidence with interpretation of data by the authors, all of whom are content experts [17].

## Results

The midlife women’s challenges revealed one overarching theme, “Searching for balance in the midst of multiple co-occurring stressors while coping with losses and transitions, for some in a context of limited resources” and five themes: 1) Changing Family relationships, 2) Re-balancing Work and Personal Life, 3) Rediscovering Self, 4) Securing Enough Resources, and 5) Coping with Multiple Co-Occurring Stressors. Each theme was further divided into categories. If a response contained more than one challenge, the challenges were each counted individually as well as placed into the Multiple Co-Occurring Stressor category. For example, if a response conveyed parenting a teenager, husband’s health, and a parent’s death, there would be three separate types of challenges as well as the Multiple Co-Occurring Stressor challenge.

### Searching for balance in the midst of multiple co-occurring stressors while coping with losses and transitions, for some in a context of limited resources

Data analysis revealed an overarching theme, “Searching for balance in the midst of multiple co-occurring stressors while coping with losses and transitions, for some in a context of limited resources,” that encapsulated the experiences of all study participants and permeated the themes and sub-themes that emerged from the data. Women reported challenges related to changing family relationships, including those with several generations of family members, e.g. children and parents, while also striving to rebalance their work and personal life. In addition, they struggled with rediscovering themselves, in the context of changing relationships. Securing sufficient financial resources posed challenges for many. A noteworthy set of challenges is related to coping with multiple co-occurring stressors. Each of these is discussed in greater detail below.

### Changing family relationships

The theme of *Changing Family Relationships* refers to the changing relationships that women had with different family members: husband/partner, children, aging parents, siblings, and in-laws.

#### Changing relationships with partner

A number of women described changes in their long-term relationship with their partners as a primary life stressor. These changes ranged from a declining partner’s health and necessity to provide caregiving, to separation or divorce after many years together, to the untimely death of a partner. Some women, especially those who reported divorce/breaking up with partner, reported more than one challenge. For example, one woman explained that an all-encompassing life challenge was a combination of events she summed up as “my divorce, my children leaving home and my parents dying all in the same 2-year period.” Like others, another woman wrote, “The death of my brother in [year] and my divorce the same year” presented the most challenge. A partner’s declining health was another new life challenge for many study participants. Women disclosed having to deal with their partners’ declining health, including heart attacks, depression, disability, surgery, high blood pressure, reluctance to be more active, and alcoholism as a challenge. Like others, one study participant explained, “The most challenging has been watching my husband sink more and more into alcoholism and not being able to stop him.” Another woman shared, “The challenges have changed from year to year- [year] I had an ectopic pregnancy- and infertility before/after- 0 kids. [years]- Graduate school and full-time work was challenging. [year]- currently, my husband’s health problems and disability are most challenging.”

For other midlife women the most challenging experiences were around the transition from an old to a new partner relationship. Similar to others, for one woman the life-midlife turmoil included “Losses and transitions –death of both parents, divorce from long term partner, beginning a new life with a new partner and his child.”

#### Changing relationships with children

For many women in the study, changes in relationships included challenges with parenting that ranged from foster-parenting, parenting step-children, leaving children, children moving back in, to children moving out (Empty Nest), death of a child, or dealing with infertility. For some of the women many of these issues were intertwined. For most women in the study, problems with parenting teenage children presented a new challenge. For others, step-parenting or foster parenting was most difficult. One woman explained, “Foster-parenting teens, most often teens who have been victims of abuse.” Another one reflected parenting step-children was a new life challenge for her. She explained, “Dealing with being a blended family. Trying to parent stepchildren who would rather not have me around…” was quite difficult. For others, dealing with more than simply parenting teens added to the complexity of the parenting experience. Like many other women, one study participant listed multiple challenges, “My current job, my daughter from age 15-18, my mother’s death, my husband’s unemployment.”

Children leaving home (e.g., for college) and children moving back home were also challenging for some women. One woman explained, “Family life – Change from having little children to them all growing up and leaving – changing relationship with husband because of that and personal changes” was strenuous. A woman whose child came back home said, “getting older, stiffer, clumsier. Seeing my finances shift, caring for 2 elderly parents and having a grown child move home w/ no finances” was wearisome. One woman in the study shared, “My son dying in [year] from suicide” was her greatest midlife challenge.

For childless women in the study, a midlife challenge represented a realization that biological parenthood will never be part of their life experience. One woman reflected, “The challenges have changed from year to year – [year] I had an ectopic pregnancy – and infertility before/after resulting in 0 kids. [years]– Graduate school and full-time work was challenging. Currently my husband’s health problems and disability is the greatest challenge.” Another woman indicated, “accepting that I would never be a biological parent, never have my ‘own’ kids, and possibly never become ‘important’ to my two step-children (now grown and living away). Everyone else’s pregnancies, baby showers, and ‘kid talk’ is also a challenge.”

#### Changing relationships with aging parents

Caregiving for parents, death of parents, parents’ health problems, and relationships with parents encompassed the women sharing about their relationships with aging parents. Like others, one woman shared, “Caregiving for parents and losses are challenging – Losing father [year], father-in-law [year], mother-in-law [year], and only having my mother still living.” For some study participants, death of a parent was the most challenging part of their midlife experience. Others described, “Losing my Dad to brain cancer,” and “Experiencing my parents’ death” as the most challenging part of midlife. One woman remembered, “Within four months, my mother had a severe stroke, my father died and a month later (to the day) my mother passed away.” For other study participants who still had their parents, “parents getting old” and “Dad’s health” were cited as the most challenging.

#### Changing sibling relationships

The issues surrounding women’s changing relationships with siblings consisted of three key narratives: death of a sibling, relationship problems with siblings, and seeking harmonious sibling relationships. Women recounted, “the death of my brother in [year] and my divorce the same year” and “Dealing with not getting along with my older sister” as some examples of the most challenging part of midlife.

#### Changing relationships with in-laws

With aging in-laws, for some women in the study challenges came from having to move in together. One woman reported, “Moving in and living with all of my In-Laws” was the most challenging thing she had to do.

### Re-balancing work and personal life

For many women in the current study, stressful job/career, unemployment, balancing multiple roles, job change/ career change, job loss/ unemployment, finding a job with health benefits, and facing retirement necessitated re-balancing work and personal life. Only three out of 81 women in the study cited their job as the most challenging part of midlife. For the majority of women, feeling overworked, and having to balance multiple roles was difficult. Like others, one woman explained: “Balancing all aspects of my life - as a mother, as a wife, as a teacher and as a woman and as the major head of the household (cooking, cleaning, etc.) currently is the greatest challenge of my life.” For others, the greatest challenges came from “Getting into a more interesting career,” dealing with personal health issues such as a breast cancer diagnosis, going through a divorce, or losing a partner, losing a job and seeking new employment with benefits. One woman elaborated,

“Finding and sustaining suitable employment with health care benefits. Having intermittent medical coverage caused me to postpone a surgery (hyperparathyroid) for 3 years” as the most significant issue she had to deal with in midlife.

### Re-discovering self

Re-discovering self was important to many women in the current study. Health problems, existential issues, self-esteem/ self-acceptance, returning to school, the menopausal transition, and personal changes were the five sub-themes related to the self. Many women commented about health problems they had. Health problems included heart surgery, arthritis, physical disability due to arthritic pains, chronic pain, breast cancer, motor vehicle accident resulting in the diminished use of the woman’s right hand, blot clot in the leg, and as one woman summarized: “getting older, stiffer, clumsier.”

Women focused on making meaning of or appraising various aspects of their lives. Some of the women focused on accepting not being able to achieve their goals in life, realizing that the number of active years is limited, others on seeking new relationships. A number of women remarked about the newly found comfort with whom they were and self-acceptance. Similar to others, one study participant concluded, “Becoming more comfortable with myself. Accepting myself & having better self-esteem…” was most challenging. In order to re-discover oneself, some women returned to graduate school or decided to finish the university degree they once started. Surprisingly, only four out of 81 women in the current study commented on their menopausal transition symptoms as being the most challenging aspect of midlife, which included hot flashes, mood swings, difficulty remembering things, and excessive uterine bleeding.

### Securing enough resources

Generating enough resources was an all-encompassing task for many study participants. The women found financial challenges, partner’s unemployment, and lack of health insurance as very stressful life issues.

Some women revealed financial challenges such as supporting children in private schools with a partner’s sporadic job situation, financing college, and becoming financially secure as stressful. Many of them described how they coped with such situations in life. For example, one woman enumerated, “I have to work 2 jobs and long hours to support my children, but never seem to get ahead…” Another woman explained, “having to close a business, including laying off people, not paying business debts, selling off furniture, etc., and then having to sell our home to pay off a bank loan” as most challenging. For some women the difficult financial decisions were related to their partner’s unemployment. One example was “…Constant threat of strikes or job lay off for my husband and eventually job loss was difficult.”

Lack of health insurance was also a great concern to women. One woman related that “Finding and sustaining suitable employment with health care benefits” and “Having intermittent medical coverage” were the greatest challenges for her.

### Coping with multiple co-occurring stressors

As stated in the preceding paragraphs many women in the current study had to deal with multiple life stressors in their midlife years, many of them occurring at or around the same time. The majority of women identified multiple co-occurring stressors as they described their most challenging experiences. One woman commented, “Dealing with stress – job stress, health stress, social stress, family stress, etc. For a time, it seemed to snowball with no end in site.” Some women explained that being overworked and balancing multiple roles were the most challenging part of midlife. Two examples were, “Fulfilling obligations of work and family” and “Balancing all aspects of my life - as a mother, as a wife, as a teacher and as a woman and as the major head of the household (cooking, cleaning, etc.).”

## Discussion

Seattle Midlife Women’s Health Study participants found themselves searching for balance in the midst of multiple co-occurring stressors while coping with losses and transitions, for some in a context of limited resources. Themes of challenges for this group of midlife women included 1) changing family relationships, 2) re-balancing work and personal life, 3) re-discovering self, 4) securing enough resources, and 5) coping with multiple co-occurring stressors.

Research about self-in-relation to others [18] provides a useful framework for understanding the salience of these categories of challenging experiences. Taking care of family members with whom women have affiliations or connections is central to the lives of many women. Women’s affiliations are organized around being able to make and maintain relationships with others. Taking care of others (partners, children, parents) is one way of describing how women’s connections are formed. For many women, the threat of terminating a connection is viewed not only as a loss of connection, but as a total loss of self. Losses were exemplified in this study as many women identified divorce and losing their parents as the most challenging aspect of midlife in *Changing family relationships*. In an ethnographic qualitative research study from Australia [19], Dare and colleagues found that while many midlife women cope with the menopausal transition and their children leaving the house, the aging and death of their parents [20] and the effect of divorce [6] present more serious long-term challenges to these women.

In addition to relational issues, the workplace continues to provide many challenges for women. Overwork is a common experience, detailed well in Hochshild’s “Second Shift” [21]. The combination of responsibilities women assume beyond their employment remain daunting for U.S. women, with many not having access to help with child care and household maintenance. Indeed, Hochshild’s observations were that women worked the equivalent of a ‘second shift’ after they returned home after employment. Thus, launching children may be emancipatory for both the late adolescent and young adult children as well as their midlife mother.

Recent study of women’s multiple roles, including work-family conflicts, has largely focused on younger, reproductive age women with preschool or school-aged children. Recognition of the continuity of the challenge of balancing competing demands of work and family for midlife women, and the addition of caring responsibilities for their parents, points to increased complexity of achieving balance and leading to the use of the term “sandwich generation” [22] to describe the compression of midlife women’s lives by their children’s and parents’ needs.

In addition to rebalancing work and personal life, women faced challenges of re-discovering themselves in the context of their changing relationships. Miller and Stiver [18], proposed that a woman’s sense of self and self-worth is often grounded in her ability to make and maintain relationships, and that these connections, not separations, can lead to strong, healthy development. Individual development, seen in the category of *Re-discovering Self*, proceeds by means of connection. Women connect with other women by finding relationships that foster mutual growth or mutuality. Mutuality benefits both people to grow and develop in and as a result of the relationship. This mutuality may manifest itself in a woman with breast cancer connecting with a breast cancer support group or with others who have a different health problem. Women may develop further by questioning their existence, their purpose, or raising other existential questions that surface in midlife and relating these questions to someone with whom they share mutuality. According to Miller and Stiver [18], the inclination toward connection that women feel in themselves is a strength. Any matter in question in relation to the self may enable women to develop themselves further by sharing with another person; this forwardness of mutuality increases the strength of the relationship.

Women’s descriptions of challenges related to re-discovering themselves reflected their interest in the next stage of their lives. Current literature about women’s experiences of aging emphasize the “third chapter:” Sarah Lawrence-Lightfoot coined this term to designate the years when one is neither old nor young, a period that can be transformative in women’s lives. In her case studies of adults, Lawrence-Lightfoot explores themes of engagement over retreat, labor over leisure, and reinvention over retirement, emphasizing the importance of active engagement, purposefulness, and new learning as themes in the stories that people write about in the third chapter of their lives [23]. Mary Catherine Bateson [24] also examines the middle years, revising Erik Erickson’s model of human development to include a second stage of adulthood in which the challenges include engagement over withdrawal. Her discussion of lifelong learning as part of human developmental processes emphasizes the achievement of wisdom and humility as one confronts the challenges of aging. Both Lawrence-Lightfoot [23] and Bateson [24] invite consideration of midlife as a period during which it is possible to actively compose a life story or narratives into which we can live as we age.

Often before a woman has the opportunity for self-introspection, she may find that dealing with concerns about material resources, such as financial worries, employment and health insurance take precedence. Although the majority of women in this study were not living in poverty, women experience disproportionately lower incomes than men. For the fourth quarter of 2017, the Bureau of Labor Statistics reported weekly median earnings for women who were full-time wage or salary workers as $771.00, which was 82% of the $944.00 median weekly earnings for men [25]. During midlife, both men and women reach their peak earning capacity. Women who leave the labor force to raise their children or those women who have been laid off, struggle with access to benefits from employment, such as healthcare and often lag behind in their cumulative retirement benefits in comparison to men. A woman’s exposure to securing enough resources is also impacted by her partner’s employment status. For example, when a woman’s partner faces unemployment, the family experiences the consequences, especially if the employment benefits, such as health insurance, are from the partner’s employer. If a woman’s income is the primary household income, job loss can also result in loss of healthcare if the healthcare benefits are from her employer.

The most commonly experienced challenges for midlife women across all themes were identified as *Coping with Multiple Co-occurring Stressors*. Midlife women reported multiple co-occurring stressors when asked what was the most challenging for them during the past 15 years. Midlife is marked by women who are overworked with multiple roles and responsibilities. Lanza di Scalea et al. [26] investigated role stress, role reward, and mental health in a cross-sectional sample of 2549 women, who were 45–55 years with roles such as being employed, married, a mother, and/or a caregiver, revealing 34% of the sample were involved in 2 roles and 50% of the sample were involved in 3 roles [26]. The roles reported [26] were similar to those reported in this study as challenges in a woman’s job, being married/partnered, being a parent, and taking care of elderly parents.

Only four women (4/81 = 5%) in the SMWHS sample reported the menopausal transition as being part of the most challenging aspects of midlife, identifying hot flashes, mood swings, difficulty remembering, and excessive uterine bleeding. Thus, their challenges were not with experiencing the menopausal transition itself, but, experiencing symptoms. This finding is surprising given that 85% of midlife women report one or more symptoms, such as hot flashes, depressed mood, and/or sleep disturbances [27]. In the Penn Ovarian Aging Study, 26 % of women disclosed moderate to severe hot flashes and 9 % revealed having daily hot flashes [28]. Also, The Study of Women’s Health Across the Nation (SWAN) identified 60–80% of women experience hot flashes at some point during the menopausal transition [29]. Despite the prevalence of symptoms, it is possible that the stressful nature of the menopausal transition has been over-emphasized [30].

This study has several limitations. First, the sample size consisted of responses from only 81 women, most of whom were White, employed, and married or partnered. The average age was 39 years at enrollment (approximately 53–54 years at the time of the current study) with an average of 17 years of education and 61% were parents. The participants included in this component of the SMWHS differed from those in the parent study who, at enrollment, were older, made less money, were more ethnically diverse, were less likely to be married, and more likely to be parents. Additionally, the women’s answers to the Health Questionnaire usually included only 2–3 sentences, and their responses to a written questionnaire vs interview made further clarification of their responses impossible. Future investigations should include more ethnic diversity. Geronimus and colleagues [31] studied weathering on Black and White adults aged 18 to 64 years using logistic regression and odds ratios and found that Black women have a larger allostatic load compared to either Black men or White women and that marked differences were between non-poor Black women and non-poor White women suggesting that race is a key component in the impact of chronic stress on health [31].

The current investigation had several strengths. This study is the first to examine midlife women’s descriptions of their challenges over an extended reference period of 15 years while participating in the Seattle Midlife Women’s Health Study. Results of this study included the most commonly reported challenges over the past 15 years of midlife explained by the women themselves. These findings are important as they reveal the challenges most salient to midlife women and may also help providers to identify women at high risk for allostatic overload (e.g., sustained high blood pressure, sustained high levels of cortisol as a result of chronic high levels of stress), which may lead to heart disease, stroke, or sleeping problems. Further, providers will find these results informative in order to individualize care, so that they can determine resources and interventions to help this specific age group of women who perform so many roles with all their associated responsibilities.

## Conclusion

The over-arching theme of searching for balance in the midst of multiple co-occurring stressors, while coping with losses and transitions, for some in a context of limited resources, spanned five categories. The most frequently reported challenges identified were multiple co-occurring stressors. Further study of multiple co-occurring stressors is warranted. Perhaps a single stressor, e.g., divorce, precipitates several related stressors. For example, loss of life partner precipitates loss of income, loss of children and separation from a relational network of mutual friends of the couple. Also, experience of a single stressor, such as development of a chronic illness, may precede other stressors such as job loss, a need to relocate living arrangements and the financial stressors of paying for medications. Inquiring about a focal stressor and its consequences may help women elaborate a series of stressors that more fully illuminates midlife women’s experiences.
